# Application of Intervention Mapping to develop a community-based health promotion pre-pregnancy intervention for adolescent girls in rural South Africa: Project Ntshembo (Hope)

**DOI:** 10.1186/1471-2458-14-S2-S5

**Published:** 2014-06-20

**Authors:** Catherine E Draper, Lisa K Micklesfield, Kathleen Kahn, Stephen M Tollman, John M Pettifor, David B Dunger, Shane A Norris

**Affiliations:** 1 MRC/Wits Developmental Pathways for Health Research Unit, University of the Witwatersrand, Johannesburg, South Africa; 2 UCT/MRC Research Unit for Exercise Science and Sports Medicine, Sports Science Institute of South Africa, Boundary Road, Newlands, Cape Town, South Africa; 3 MRC/Wits Rural Public Health and Health Transitions Research Unit (Agincourt), School of Public Health, University of the Witwatersrand, Johannesburg, South Africa; 4 Department of Paediatrics, Addenbrooke’s Hospital, University of Cambridge, Cambridge, United Kingdom

## Abstract

**Background:**

South Africa (SA) is undergoing multiple transitions with an increasing burden of non-communicable diseases and high levels of overweight and obesity in adolescent girls and women. Adolescence is key to addressing trans-generational risk and a window of opportunity to intervene and positively impact on individuals’ health trajectories into adulthood. Using Intervention Mapping (IM), this paper describes the development of the Ntshembo intervention, which is intended to improve the health and well-being of adolescent girls in order to limit the inter-generational transfer of risk of metabolic disease, in particular diabetes risk.

**Methods:**

This paper describes the application of the first four steps of IM. Evidence is provided to support the selection of four key behavioural objectives: viz. to eat a healthy, balanced diet, increase physical activity, reduce sedentary behaviour, and promote reproductive health. Appropriate behaviour change techniques are suggested and a theoretical framework outlining components of relevant behaviour change theories is presented. It is proposed that the Ntshembo intervention will be community-based, including specialist adolescent community health workers who will deliver a complex intervention comprising of individual, peer, family and community mobilisation components.

**Conclusions:**

The Ntshembo intervention is novel, both in SA and globally, as it is: (1) based on strong evidence, extensive formative work and best practice from evaluated interventions; (2) combines theory with evidence to inform intervention components; (3) includes multiple domains of influence (community through to the individual); (4) focuses on an at-risk target group; and (5) embeds within existing and planned health service priorities in SA.

## Background

Sub-Saharan Africa is undergoing multiple transitions with rapid urbanisation and economic development leading to changes in lifestyle and dietary habits [1]. One of the consequences, which partly accounts for the increase in the incidence of non-communicable diseases [2], is the double burden of early life under-nutrition, characterised by low birth weight and postnatal stunting, and later over-nutrition characterised by rapid child and adolescent weight gain and obesity [3]. South Africa (SA) is one of the African countries further along in this transition [4], where the increasing burden of non-communicable diseases (NCDs) in both rural and urban areas has been reported, with steady increases in type 2 diabetes (T2D), hypertension and ill-defined heart disease [2]. In SA between 1999 and 2006, premature adult deaths from diabetes increased by 38% among adults [2], and it has been proposed in these transitioning populations that T2D may exceed the public health burden from communicable diseases such as HIV [5,6].

Furthermore, the results of the first South African National Health and Nutrition Examination Survey (SANHANES-1), released in August 2013, put the combined prevalence of overweight and obesity in women (15 years and older) at 65%, and at 77% for women between 45-54 years of age [7]. These high levels of female overweight and obesity have serious implications for the intergenerational transfer of metabolic disease risk, particularly with regard to altered maternal glucose metabolism during pregnancy and the related consequences for the infant [6,8]. High maternal body mass index (BMI) and gestational diabetes mellitus (GDM) are independent risk factors for perinatal complications, and GDM has been independently associated with an increased risk of neonatal morbidities [9]. Children of mothers who had GDM during pregnancy are also at increased risk for obesity and T2D [10].

In light of these risks, preconception care is crucial, and optimising health in the pre-pregnancy period is especially important for glycaemic control [11]. However, many adolescent girls and women in low- and middle-income countries (LMICs) do not have access to preconception care, and there has been a call to broaden the reach of current preconception interventions, particularly those that are cost-effective, in these settings [12]. The pregnancy period presents another opportunity for intervention, and there is some evidence that indicates that women improve health behaviours, such as decreasing smoking, alcohol and caffeine consumption during pregnancy [13].

Recent unpublished data from a rural health surveillance site in SA [14] indicates that 65% of women in this area have had a child by the age of 20. Considering the high proportion of women who have had a child by age 20 years of age in SA, it is essential that pre-pregnancy interventions target adolescents to minimise transgenerational risk. Furthermore, the importance of preconception interventions to promote adolescent health has recently been emphasised, particularly in countries with a young age at first pregnancy and where the prevalence of under-nutrition is high [6,15]. In addition, the increasing burden of non-communicable diseases among adolescents has been noted [16], and Sub-Saharan Africa has been reported to have the worst regional health profile for adolescents [17]. In SA there are concerning rates of overweight and obesity amongst adolescent girls (29% overweight and 7.5% obese in 2008), with higher rates amongst urban compared to rural adolescents [18]. Unsafe sexual behaviour amongst adolescents is also a pertinent and concerning issue in SA as it increases adolescents’ exposure to HIV/AIDS and sexually transmitted infections, and contributes to the incidence of teenage pregnancy [19].

According to Dean et al [12] it is essential that adolescent health and reproductive health be regarded as critical stages in the continuum of care. They have advocated a systems-based approach to expand the demand for and uptake of preconception interventions, especially by adolescents, and support task-shifting to community health workers as part of this systems-based approach [12]. Hanson et al. [6] have argued that preconception interventions with adolescents should include a health literacy component, which involves increasing access to health information, and development of the capacity to use this information effectively.

In addition to being an optimal life stage for preconception interventions, adolescence has been recognised as a key development phase for the establishment of future patterns of adult health [20], presenting an opportunity to intervene and positively impact on individuals’ health trajectories into adulthood. According to Viner, it is a “second sensitive developmental period in which puberty and rapid brain maturation lead to new sets of behaviours and capacities that trigger or enable transitions in family, peer, and educational domains, and in health behaviours. These transitions modify childhood trajectories towards health and wellbeing” [21].

As highlighted by studies in both urban and rural SA, a major challenge is the complex interrelationship between poor maternal nutrition and postnatal stunting on one hand, and the increased risk of adolescent and adult obesity on the other, leading to an increased risk of T2D and metabolic disease in future generations, which is further accelerated by weight gain. **The primary hypothesis** driving the proposed intervention presented in this paper is that maternal pre-pregnancy body composition, pregnancy weight gain, and altered maternal glucose metabolism, can significantly impact maternal postnatal risk of T2D, pregnancy complications, offspring growth and body composition, and later risk for T2D and metabolic disease in the offspring.

### Aim

This paper describes the application of the Intervention Mapping approach to the development of an intervention, referred to as the Ntshembo intervention, which is intended to improve the health and well-being of adolescent girls in order to limit the intergenerational transfer of risk of metabolic disease. Due to the double burden of under- and over-nutrition in SA and its concomitant risks, the intervention aims to optimise pre-pregnancy health (minimise under- and over-nutrition).

## Methods

### Study (intervention) setting

The work presented in this paper forms part of a broader body of work referred to as: “Project Ntshembo – Health and well-being of female adolescents: limiting the inter-generational risk of metabolic disease”. The word ‘Ntshembo’ means ‘hope’ in Shangaan, one of SA’s official languages, and the main local language used in the study area, with the connotation around hope for a healthier future. The project started in 2007 with collaborating institutions that included the University of the Witwatersrand (Wits, SA; leading institution), University of Cambridge (UK), University of North Carolina at Chapel Hill (USA), University of Oxford (UK), Umea University (Sweden), University of Southampton (UK), and the University of Cape Town (SA).

The **primary hypothesis** is that maternal pre-pregnancy body composition, pregnancy weight gain, and altered maternal glucose metabolism, can significantly impact maternal postnatal risk of T2D, pregnancy complications (such as obstructive labour), and offspring growth, body composition and later risk for T2D and metabolic disease. Therefore, the intervention aims to reduce the trans-generational risk of T2D. For the purpose of this project, adolescence refers to the period between 13-19 years of age.

The rural study site for Ntshembo is the Agincourt sub-district of Bushbuckridge, Mpumalanga Province, near the Mozambique border where the MRC/Wits Rural Public Health and Transitions Research Unit (Agincourt) leads a health and socio-demographic surveillance system [14]. The Agincourt area has a population density of ±170 persons per km^2^, and is characterised by household plots supporting limited subsistence agriculture. Unemployment is widespread, with an estimated 60% of men and increasing numbers of women migrating to more urban areas for work, and many households dependent on social grants, such as old age pension and child support grants [22].

Formative work in Agincourt has identified an “explosive combination of early childhood stunting and adolescent obesity”, as well as a shift in child growth and nutrition towards a more urban profile [3]. The prevalence of stunting is 18% among children aged one to four years, with a peak at 32% in infants aged one year. The prevalence of overweight/obesity across the adolescent years is significantly higher in females (15%) than in males (4%) and the prevalence of overweight/obesity in adolescent females increases with age and peaks at 25% at 18 years. A similar trend was observed with central obesity with 35% of 18 year old females having a waist circumference greater than the recommended adult cut-off of ≥80cm [3]. HIV prevalence in persons 15 years and older in Agincourt is 23.9%, and 5.5% among females in the 15-19 year old category [23].

The work of Ntshembo has been embedded within the existing community engagement processes followed within Agincourt, which are coordinated through the ‘Learning, Information Dissemination and Networking with the Community’ (LINC) office within the Agincourt Research Unit. This involves a formal process of ‘community entry’, which includes consultation with local stakeholders and community leaders, and helps to highlight community needs and priorities. This was intended to create a greater sense of community ownership of and participation in the work of Ntshembo, and will play a crucial role in future intervention development work. It is essential that the Ntshembo intervention is responsive to community needs and priorities, and that the intervention activities are feasible and sustainable in a rural setting such as Agincourt.

### Intervention Mapping (IM)

The Intervention Mapping (IM) approach provides a framework for the development of health promotion programmes [24], emphasising the importance of theory and evidence in programme planning. IM ascribes to a social ecological paradigm, acknowledging that programmes can target behavioural and/or environmental factors, and that determinants of health are both personal and environmental.

The IM process involves six steps: 1) needs assessment, 2) formulation of change objectives (intervention objectives and their determinants), 3) selection of theory-based methods and practical strategies, 4) intervention development, 5) development of adoption and implementation plan, and 6) evaluation planning. This paper will focus on steps 1-4, as these represent the progress made on the project thus far. The IM process aligns with the UK MRC guidelines for the development and evaluation of complex health promotion interventions [25]. In particular, both emphasise the need to take both evidence and theory into consideration. The focus on both personal and environmental determinants within IM increases the complexity of a health promotion intervention, therefore requiring a strategy for developing and evaluating such an intervention.

The needs assessment in IM is based on a modified PRECEDE model. The PRECEDE-PROCEED model and other approaches were considered as possible frameworks for intervention development. However, the authors believed that the IM approach provides the most comprehensive approach to evidence- and theory-based intervention development that specifies the processes involved in programme design and development, and therefore opted for the use of this framework. The IM approach has been used in the development of a number of health promotion interventions, and has been described as a helpful approach for the systematic development of these interventions [26-31]. Strengths of the IM approach include its ability to take into account the complexity of intervention research [26,29], the way in which it can lead to a coherent intervention model [28], and the extent to which the approach ensures that interventions are grounded in theory and evidence [29]. Furthermore, the IM approach helps to tailor an intervention to suit the needs of a specific population [29]. While the IM approach provides a fairly structured process, some have used it in a more iterative manner [27,28], and the need for flexibility in the approach is acknowledged by its authors [24].

### Step 1: needs assessment

The needs assessment, using IM, involves an analysis of the health problem, associated behavioural and environmental conditions, and determinants of these conditions for the population at risk. In this paper, the evidence reviewed for the needs assessment includes international research on the health problem (and associated conditions and determinants) as well as research conducted in SA. A large proportion of the SA literature to be presented (published and unpublished) relates to the communities where the intervention is intended to be implemented, and this formative work has been conducted by the MRC/Wits Developmental Pathways for Health Research Unit, the MRC/Wits Rural Public Health and Health Transitions Unit (Agincourt) and the School of Public Health, University of the Witwatersrand. Furthermore, some of this data draws from the Birth to Twenty cohort study in Soweto, Johannesburg, that has been following participants for over 23 years since their birth in 1990 [32].

While there is likely to be variability between SA communities, the more general SA data helps to provide the necessary local context within the international literature that will be presented. The section that follows provides the best available balance of community-level, national and international evidence, and meets the requirements of a needs assessment within the IM approach.

#### Reviewing the evidence

##### Reproductive health

Despite a reported decline in teenage pregnancy in SA, the percentage of adolescents who report having been pregnant is still high, at approximately 30% [33], and the influence of social and gender issues has been acknowledged [34]. Among female adolescents (13-18 years old) in SA, 30.2% have had sex [35]. In Agincourt, qualitative work on adolescents’ cultural beliefs and practices indicates that adolescents know about risks and the need for protection from HIV and pregnancy, but that they need people to talk to about real life issues, e.g. dating, peer pressure and sexual risk taking, and have reservations about going to the clinic for contraception [36].

It is important to note that the Ntshembo intervention will not seek to directly address risk behaviours that lead to teenage pregnancy, such as unprotected sex, since these behaviours have been the focus of other interventions in SA, many of which have a primary focus on the prevention of HIV. The most well known of these is a national youth HIV prevention intervention, loveLife [37]. The intervention will address HIV risk, health service access around reproductive health, and planned pregnancy.

##### NCDs, overweight and obesity

Risk factors for NCDs, such as physical inactivity, unhealthy diet and obesity [38], have been shown to be prevalent amongst youth in SA [35], and the concerning rates of overweight and obesity amongst SA girls have already been mentioned [18]. In addition, there is a higher prevalence of overweight and obesity in girls than boys [3,39,40], and overweight and obesity have been associated with higher socioeconomic status (SES) in SA adolescents [18,40]. Other research has identified overweight and obesity, as well as physical inactivity and diet as important risk factors for NCDS in 15-24 year old SA females, particularly in urban areas [41]. These findings highlight the need to optimise body composition before and during pregnancy in adolescent girls in order to reduce the intergenerational transfer of metabolic risk.

Although there is limited data from rural settings, and although adolescent obesity has been shown to be higher in urban compared to rural adolescents, the increase in adolescent obesity in girls from rural settings is concerning, as is the shift in child growth and nutrition towards a more urban profile in rural areas, as mentioned earlier [3]. The urban data presented here is therefore applicable when considering adolescent obesity and health behaviours in rural settings.

Regarding determinants of obesity, a complex array of socio-cultural and environmental factors has been associated with obesity in black SA women [42], and a higher tolerance of a larger body size amongst black girls [43-45] has been linked to social and cultural norms [46]. In terms of family influences, an authoritative parenting style has been associated with lower levels of obesity [47].

##### Unhealthy diet

SA is said to be undergoing a nutrition transition, which involves a shift towards a diet high in fat, sugar and refined foods [4,39]. Research has shown that SA adolescents follow a typically Western diet that is high in fat [48] and starch [49], and includes a high intake of fast foods from commercial and informal outlets [39,50], and low fruit and vegetable consumption [49,50]. Another SA study found that adolescents brought more unhealthy than healthy items to school, and the items they bought at school were predominantly unhealthy [51]. Studies with urban and rural (Agincourt) adolescent girls in SA have identified barriers to healthy eating practices, and these include persisting poverty and food insecurity, particularly regarding the affordability and accessibility of healthy food, as well as aspirations to purchase fast foods as they are perceived to be more socially desirable [50,52]. These studies also found that knowledge of healthy dietary practices was not deficient amongst adolescent girls, but that a disparity existed between this knowledge and their healthy dietary practices [50,52]. However, other research with SA adolescents found an association between poor knowledge of dietary fat and the consumption of an unhealthy diet [48].

In young SA adults, consumption of fast foods has been associated with lower SES [53]. Parents’ healthy eating behaviours [27,54-56] (with a stronger association in LMICs compared to high-income countries [57]), an authoritative parenting style [54,55,58,59], and the frequency of family meals [55,60,61] have all been associated with healthy eating amongst adolescents. Unhealthy joint decisions with peers have been associated with unhealthy dietary habits amongst adolescents [62].

##### Physical activity

In girls, physical activity (PA) has been shown to decline in adolescence [63-65], and lower activity levels have been associated with living in more urbanised areas [41,66]. Data from Australia has recognised active commuting as an important source of habitual PA in adolescent girls [67]. Although active commuting has been associated with lower SES in girls [68,69], it is a highly variable behaviour that is sensitive to change in response to external factors [67].

In a study on physical activity amongst adolescents in Agincourt, 92% reported that they walked for transport [70]. In this study, moderate-vigorous physical activity (MVPA) was found to be higher in adolescent boys than girls, and lower socioeconomic status (at the maternal, household and community levels) was significantly associated with lower MVPA and more walking for transport.

A wide range of determinants of PA for adolescents has been identified. These include personal determinants such as planning, attitude, self-efficacy, goal orientation / motivation, and participation in physical education or school sports [71]. External determinants that have been associated with high physical activity include an authoritative parenting style [59], parent support [54,55], parent education, parental PA, friend support, SES, exposure to outdoors, and involvement in available activities [72-74].

Qualitative work with adolescent girls in Agincourt has identified conflicting ideals regarding body shape (“with or without curves”) as an inhibiting factor for physical activity in this group [75]. In addition, human, financial and physical infrastructural limitations facing schools and youth groups were also highlighted as barriers to physical activity for adolescent girls living in Agincourt.

##### Sedentary behaviour

Sedentary behaviour is independent from physical inactivity, and refers to time spent sitting or lying down, and typically includes television viewing and time spent using electronic devices (screen time). Amongst girls, sedentary behaviour in leisure time has been shown to increase significantly between early- and mid-adolescence, particularly on weekends [76]. High levels of sedentary behaviour, such as more than two hours of television viewing, have been associated with poor physical and psychosocial health outcomes in children and adolescents (5-17 years), with a decrease in sedentary time associated with a decrease in BMI [77]. In 11-year old children, the prevalence of sedentary behaviour has been negatively associated with well-being, and positively associated with higher SES and weight status [68].

Gordon-Larsen et al. have argued that sedentary behaviour (which they have referred to as inactivity) has different determinants to physical activity [73]. While the evidence for these determinants is limited [71], parental education and socioeconomic status [73,74], as well as an authoritative parenting style [59] have been inversely associated with sedentary behaviour. In contrast to these findings, which are predominantly from urban settings, lower SES (at the maternal, household and community levels) has been significantly associated with less sedentary time amongst adolescents in Agincourt [70].

#### Key behavioural objectives

The evidence presented in the needs assessment provides a justification for the development of an intervention for rural-dwelling adolescent girls that would promote healthy lifestyles, optimise pre-pregnancy health (minimise under and over-nutrition) and increase the likelihood of a healthy pregnancy, should they become pregnant. The following key behavioural objectives were therefore selected for this intervention, focussing on the behaviour of adolescent girls:

• Improve reproductive health,

• Eat a healthy, balanced diet,

• Increase physical activity,

• Reduce sedentary behaviour.

### Step 2: matrices of change objectives

In this step, each behavioural objective is broken down into performance objectives, and personal and external determinants are specified for each performance objective. Combined, these performance objectives and determinants constitute change objectives, and these are mapped out systematically into a matrix for each behavioural objective. The performance objectives selected for this intervention are those considered to be the most appropriate and relevant for adolescent girls that would be targeted by the Ntshembo intervention. Literature presented in the needs assessment helped to inform the selection of determinants for these performance objectives, along with constructs from behaviour change theories (self-efficacy, for example), as well as other studies that have used the IM approach for intervention development [28]. Authors gave input on the selection of these determinants, based on their research experience.

Matrices of change objectives for the Ntshembo intervention are presented in Tables 1, 2, 3, 4, 5, 6, 7, 8, and outline the component behaviours for each behavioural objective, along with the personal and external determinants for each component behaviour. It should be noted that these tables are not a list of possible interventions, nor are they intended to provide details of the interventions. These matrices of change help to provide a ‘map’ for the development of specific intervention components.

**Table 1:** Behavioural objective – Improve reproductive health

***PERSONAL DETERMINANTS***				
**Performance objective**	**Literacy**	**Self-awareness**	**Self-efficacy**	**Personal preference**

***Know HIV status***	Understand the importance of knowing your HIV status Understanding of HIV treatment options	Awareness of HIV status	Confidence to take HIV test Confidence to seek appropriate treatment if test is positive	Choice to take HIV test

**Performance objective**	**Literacy**	**Self-awareness**	**Self-efficacy**	**Personal preference**

***Access adolescent health services***	Understanding of the conditions for which health services should be consulted Understanding of the services (including health promotion) offered by community health facilities for adolescents	Awareness of appropriate adolescent health services in the community	Confidence to consult adolescent health services in the community	Choice to consult an appropriate adolescent health service in their community

**Table 2:** Behavioural objective – Improve reproductive health

***EXTERNAL DETERMINANTS***			
**Performance objective**	**Social norms**	**Social support**	**Resources and services**

***Know HIV status***	Peers are perceived to know their HIV status	Peers and family support knowing HIV status	Availability of HIV voluntary counselling and testing services for adolescents

**Performance objective**	**Social norms**	**Social support**	**Resources and services**

***Access adolescent health services***	Peers are perceived to be actively seeking appropriate adolescent health services, including health promotion services	Peers and family support the seeking of appropriate adolescent health services	Availability and accessibility of appropriate adolescent health services Availability of appropriate health care workers trained to deliver health promotion (including education) and treatment to adolescents

**Table 3:** Behavioural objective – Eat a healthy, balanced diet

***PERSONAL DETERMINANTS***				
**Performance objective**	**Literacy**	**Self-awareness**	**Self-efficacy**	**Personal preference**

***Reduce consumption of sugar*** **(*****e.g. sugar-sweetened beverages*****, *****sweets*****,** ***etc.*****)**	Understanding of the importance of limiting sugar intake	Awareness of sugar consumption habits Awareness of healthy alternatives to high sugar foods and beverages	Confidence to reduce intake of sugar and choose healthy alternatives, especially in social settings	Selection of healthy alternatives to high sugar foods and beverages

**Performance objective**	**Literacy**	**Self-awareness**	**Self-efficacy**	**Personal preference**

***Reduce portion size of staple starchy foods*** **(*****e.g. maize meal*****,** ***potatoes*****,** ***white bread*****,** ***rice*****,** ***pasta*****)**	Understanding of the balance of macronutrients in the diet	Awareness of portion sizes	Confidence to reduce portion sizes, especially in social settings	Selection of reduced portion of staple starchy foods

**Performance objective**	**Literacy**	**Self-awareness**	**Self-efficacy**	**Personal preference**

***Reduce the consumption of convenience foods***	Understanding of the risks if regularly eating convenience foods	Awareness of convenience food purchasing habits Awareness of healthy alternatives to convenience foods	Confidence to reduce consumption of convenience foods, especially in social settings	Selection of healthy alternatives to convenience foods

**Performance objective**	**Literacy**	**Self-awareness**	**Self-efficacy**	**Personal preference**

***Increase consumption of fruits and vegetables***	Understanding of the importance of fruit and vegetable consumption for the promotion of health	Awareness of options for purchasing fruit and vegetables	Confidence to increase consumption of fruit and vegetables	Choice to eat fruits and vegetables that they prefer

**Table 4:** Behavioural objective – Eat a healthy, balanced diet

***EXTERNAL DETERMINANTS***			
**Performance objective**	**Social norms**	**Social support**	**Resources and services**

***Reduce consumption of sugar*** **(*****e.g. sugar-sweetened beverages*****,** ***sweets*****,** ***etc.*****)**	Peers and family are perceived to be consuming less sugar Association between sugar and positive experiences is not reinforced	Peers and family support reduction in consumption of sugar	Accessibility of healthy alternatives to high sugar foods and beverages Availability of adolescent-friendly health education material, delivered by an appropriate health care worker

**Performance objective**	**Social norms**	**Social support**	**Resources and services**

***Reduce portion size of staple starchy foods*** **(*****e.g. maize meal*****,** ***potatoes*****, *****white bread*****,** ***rice*****,** ***pasta*****)**	Peers and family are perceived to be reducing portion sizes Association between large portion sizes and positive experiences is not reinforced	Peers and family support reduction in portion sizes	Availability of adolescent-friendly health education material, delivered by an appropriate health care worker

**Performance objective**	**Social norms**	**Social support**	**Resources and services**

***Reduce the consumption of convenience foods***	Peers and family are perceived to be consuming less convenience foods Association between convenience foods and positive experiences is not reinforced	Peers and family support reduction in consumption of convenience foods	Accessibility of healthy alternatives to convenient foods Availability of adolescent-friendly health education material, delivered by an appropriate health care worker

**Performance objective**	**Social norms**	**Social support**	**Resources and services**

***Increase consumption of fruits and vegetables***	Peers and family are perceived to be consuming more fruits and vegetables	Peers and family support an increase in consumption of fruits and vegetables	Accessibility of affordable fruit and vegetables Availability of adolescent-friendly health education material, delivered by an appropriate health care worker

**Table 5:** Behavioural objective – Increase physical activity

***PERSONAL DETERMINANTS***				
**Performance objective**	**Literacy**	**Self-awareness**	**Self-efficacy**	**Personal preference**

***Increase intensity of walking***	Understanding of the importance of exercise intensity in order to achieve health benefits	Awareness of walking intensity	Confidence to increase walking intensity	Choice to increase walking intensity

**Performance objective**	**Literacy**	**Self-awareness**	**Self-efficacy**	**Personal preference**

***Increase participation in community-based activities that promote movement*****, *****e.g. dancing*****,** ***sport***	Understanding of the benefits of physical activity	Awareness of available community-based activities that promote movement	Confidence to participate in community-based activities	Choice to participate in community-based activities that promote movement

**Table 6:** Behavioural objective – Increase physical activity

***EXTERNAL DETERMINANTS***			
**Performance objective**	**Social norms**	**Social support**	**Resources and services**

***Increase intensity of walking***	Walking for transport is not stigmatised Peers and family are perceived to be increasing their walking intensity	Peers and family are perceived to have a positive perception of walking Peers and family support increased walking intensity	Availability of adolescent-friendly health education material, delivered by an appropriate health care worker

**Performance objective**	**Social norms**	**Social support**	**Resources and services**

***Increase participation in community-based activities that promote movement*****, *****e.g. dancing*****,** ***sport***	Peers are perceived to be participating in community-based activities that promote movement	Peers and family support increased participation in community-based activities that promote movement	Accessibility of appropriate activities that promote movement for adolescent girls Availability of adolescent-friendly health education material, delivered by an appropriate health care worker

**Table 7:** Behavioural objective – Reduce sedentary behaviour

***PERSONAL DETERMINANTS***				
**Performance objective**	**Literacy**	**Self-awareness**	**Self-efficacy**	**Personal preference**

***Reduce screen time*** **(*****TV and computer*****)** ***to*** **<** ***2 hours per day***	Understanding of the health risks of prolonged screen time	Aware of screen time behaviour patterns	Confidence to reduce screen time	Choice to reduce screen time

**Performance objective**	**Literacy**	**Self-awareness**	**Self-efficacy**	**Personal preference**

***Select feasible active alternatives to screen time***	Understanding of the importance of replacing screen time with active behaviour	Aware of active alternatives to screen time	Confidence to engage in active alternatives	Choice of active alternatives

**Table 8:** Behavioural objective – Reduce sedentary behaviour

***EXTERNAL DETERMINANTS***			
**Performance objective**	**Social norms**	**Social support**	**Resources and services**

***Reduce screen time*** **(*****TV and computer*****)** ***to*** **<** ***2 hours per day***	Peers and family are perceived to be reducing screen time	Peers and family support the reduction of screen time	Availability of adolescent-friendly health education material, delivered by an appropriate health care worker

**Performance objective**	**Social norms**	**Social support**	**Resources and services**

***Select feasible active alternatives to screen time***	Peers and family are perceived to be engaging in active alternatives	Peers and family support active alternatives	Availability of alternative active leisure opportunities Availability of adolescent-friendly health education material, delivered by an appropriate health care worker

### Step 3: selection of methods and strategies

In step 3, theory-based methods and practical strategies are identified that will be appropriate for bringing about behaviour change in an intervention target group, which in this case is adolescent girls. Appropriate behaviour change techniques (BCTs) for the Ntshembo intervention, listed in Table 9, have been identified from the taxonomy of BCTs developed by Abraham and Michie [78], and draw on social cognitive theory [79], the theory of planned behaviour [80], control theory [81] and the information-motivation-behavioural skills model [82].

**Table 9:** Ntshembo behaviour change techniques

**Behaviour change techniques** (Abraham 2008)	Determinants
Provide information about behaviour-health link (IMB) Provide information on consequences (TPB)	Health literacy

Prompt barrier identification (SCT) Prompt specific goal setting (CT) Prompt self-monitoring of behaviour (CT)	Self-awareness

Provide general encouragement (SCT) Model or demonstrate the behaviour (SCT) Provide feedback on performance (CT) Plan social support or social change	Social support

Prompt intention formation (TPB, SCT, IMB)	N/A

Note: IMB = Information-motivation-behavioural skills model, TPB = Theory of planned behaviour, SCT = Social cognitive theory

**Figure 1:**
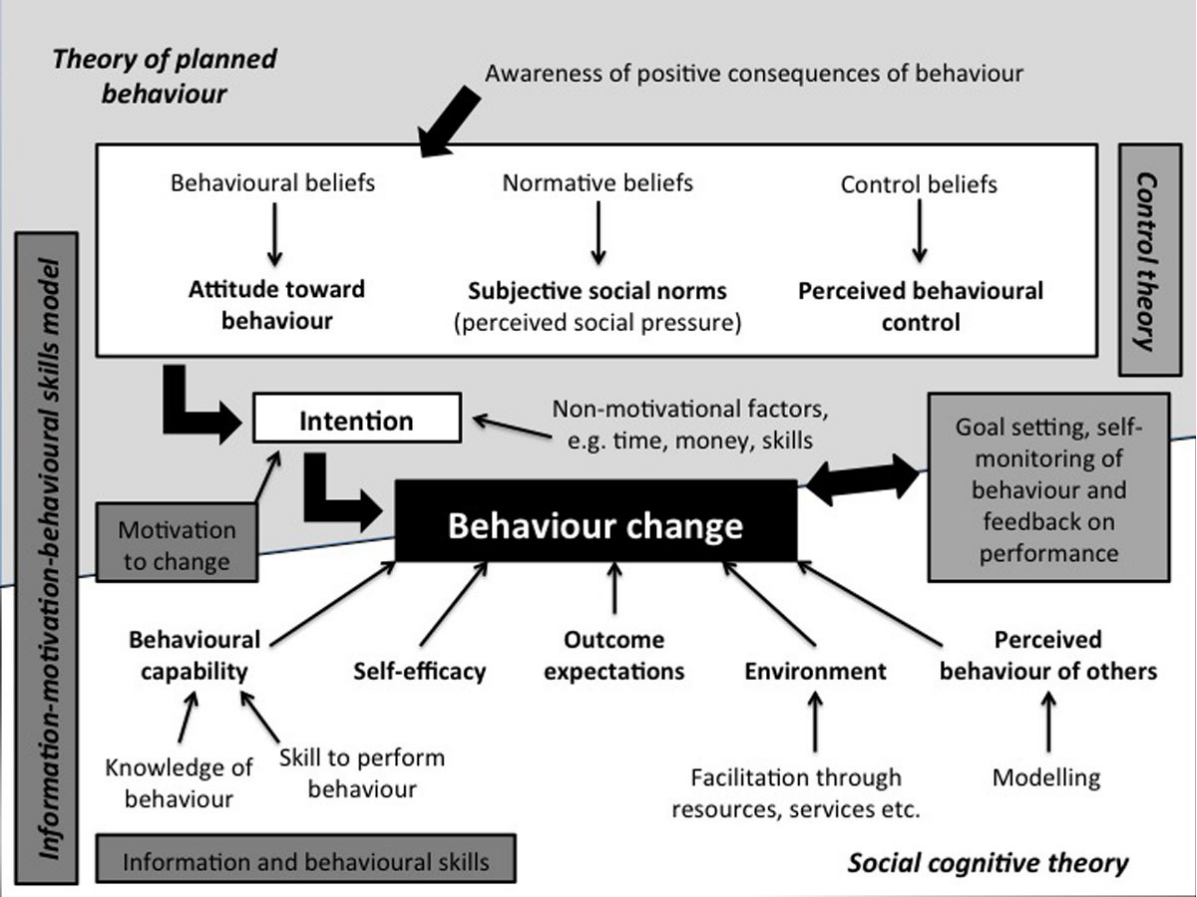
Ntshembo theoretical framework

A theoretical framework outlining the relevant components of these theories is presented in Figure 1. This framework highlights the complexities of behaviour change, illustrating the various factors that influence motivation and intention to change behaviour, and behaviour change itself. These factors include knowledge and skills to perform a behaviour; perceptions of others’ behaviour and what is considered socially acceptable behaviour; attitudes and beliefs regarding a particular behaviour and an individual’s perceptions of the extent to which they are capable of changing this behaviour.

### Step 4: intervention development

#### Evidence of intervention effectiveness

Before providing an outline of the Ntshembo intervention, it is helpful to consider evidence regarding the effectiveness of interventions for adolescent girls that optimise health. The evidence of effectiveness of interventions to promote reproductive health is limited. While early presentation for antenatal care is recommended in order to identify risks, little is known about how to effectively promote this in adult women [83,84], let alone adolescents, although community engagement programmes are recommended to enable women to seek antenatal care early in their pregnancy [83]. A lack of understanding regarding the benefits of early antenatal care has been identified as a barrier to early initiation of antenatal care [83,85].

In terms of interventions that address behaviours associated with over- and under-nutrition, school-based, multi-component interventions with adolescent girls, targeting lifestyle behaviours associated with obesity, have been shown to increase PA [86] and decrease sedentary behaviour [87], as well as improve selected body composition measures and some dietary behaviours [88]. However, systematic reviews on obesity interventions in adolescents have shown that the effectiveness of school-based interventions can be limited (only 41% of studies reviewed showed a positive effect) [89], and that there is a need for more quality trials examining physical activity behaviour change strategies appropriate for adolescents [90]. Furthermore, it is the experience of the authors that intervening in the school environment in SA is particularly challenging, especially since local and national ministries of education have become far stricter about the implementation of interventions within the school environment, even outside of learning time. This would have serious implications for the long-term sustainability of an intervention.

Community-based interventions for adolescents have been shown to improve weight outcomes [91,92] and various psychosocial outcomes [92], although challenges have been experienced in the implementation of these types of interventions [93]. Interventions aimed at obesity prevention and treatment that involve family members have been shown to be most effective [94,95], and interventions emphasising a decrease in sedentary behaviour have been shown to effectively reduce this behaviour and manage weight [96].

With regards to adolescents’ interactions with health services in SA, there have been reports of nurses’ poor treatment of sexually active adolescents [97,98]. Adolescent-friendly health services have been developed in order to address these concerns, and while adolescents use these services [99], which have been shown to improve the quality of care delivered to adolescents [100], teenage pregnancies and sexually transmitted infections have not been reduced [99]. Formative work in Soweto, Johannesburg where Youth Friendly Services have been implemented, has shown that youth do not perceive general services as being youth-friendly [101], and are often not aware of these services [102].

Community health workers (CHWs) present an alternative for delivering adolescent health services, and some have argued for CHWs who specialise in adolescent health [103]. Specialist CHWs may be able to increase their coverage by specifically targeting households with adolescents, they could help to increase awareness of adolescent-friendly health services, they could focus on key messages for adolescents to enhance behaviour change, and could develop a high level of expertise in adolescent health. More evidence is however required to compare the effectiveness of generalist versus specialist CHWs [103].

#### Ntshembo intervention

The Ntshembo intervention will be a community-based intervention that will target all adolescent girls within communities selected for the intervention, as opposed to only targeting those most at risk. Considering that the key behaviours to be addressed by this intervention relate to reproductive health, diet and activity patterns, it is possible that the adolescent girls targeted by this intervention may be at risk because of behaviours in one or more of these areas, or because of their body composition, or may not be at risk at all. The intervention is therefore intended to be flexible in its approach by focussing, at an individual level, on the most relevant area of risk (if any) for each participant.

The intervention will include specialist adolescent CHWs, which is in alignment with the SA National Department of Health’s inclusion of CHWs as an integral part of primary health care ‘re-engineering’ in SA. While the National Department of Health has not advocated the inclusion of specialist CHWs, the Ntshembo project could provide a strong rationale for the inclusion of adolescent CHWs into SA’s primary health care system in the future, especially in rural areas where the geographical accessibility of health services remains a challenge. These adolescent CHWs will be the main mode of delivery for the Ntshembo intervention.

**Figure 2:**
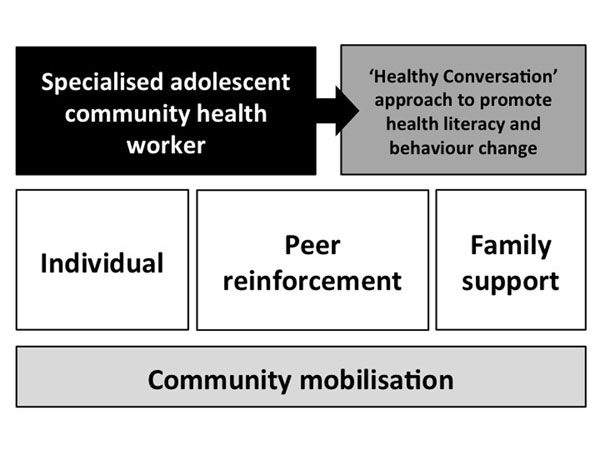
Ntshembo intervention outline

Based on the identification of personal and external determinants identified in the IM process, and in order to provide social support and facilitate the impact on social norms within the target communities, it is essential that the adolescent CHWs have contact with the individual adolescents, as well as with peers and family members. Furthermore, taking into consideration the role of a facilitating environment highlighted in social cognitive theory, the Ntshembo intervention will include a community mobilisation component. The individual, peer, family and community components are outlined in Figure 2. The combination of all these components, rather than the individual components themselves, makes the Ntshembo intervention novel.

The ‘Healthy Conversation Skills’ (HCS) approach, developed in Southampton, United Kingdom, is a training intervention for use with socioeconomically disadvantaged women. Drawing on social cognitive theory, the intention is to improve self-efficacy and perceived control in both the staff delivering the intervention and recipients of the intervention [104,105]. The HCS approach will be used by adolescent CHWs in their interactions with individual adolescents, adolescents’ family members and peer groups. It aligns well with the theoretical framework and behaviour change techniques listed earlier and presented in Figure 1.

Based on the evidence presented, as well as the IM process, an intervention plan was developed by a small working group (CD, LM and SN), and presented to the co-authors, along with the results of the IM process and a rationale for a rural adolescent health promotion intervention in May 2013. These presentations were then refined and presented to a stakeholder group in Agincourt for their comment and input in July 2013. The stakeholder group included the University of the Witwatersrand staff involved in research and community engagement in Agincourt, a local religious leader, as well as representatives of the local educational services, health services and youth organisations. Feedback from stakeholders was positive, particularly around the inclusion of adolescent CHWs. Stakeholders perceived adolescents to be reluctant to access local clinic health services because of the issues mentioned earlier, as well as the fact that staff at health services are often known by the adolescents caregivers, which may compromise confidentiality. Stakeholders also discussed cultural perceptions regarding female body size, and these will need to be an important consideration in the Ntshembo intervention.

Following the Agincourt stakeholder meeting the intervention was refined, and in August 2013 a reworked intervention was presented to another group of stakeholders that included academics, paediatricians, public health specialists, policy experts, and economists. The complex intervention was seen to be innovative, with importance to primary prevention and the Department of Health CHW framework, and relevant to policy leaders.

### Next steps

The UK MRC guidelines highlight the need for feasibility testing in the intervention development-evaluation-implementation process [25]. Based on stakeholder input, intervention materials will be developed, and these materials will undergo feasibility testing in 2014 to: (1) assess the recruitment and training of CHWs to deliver the intervention; (2) assess the content and appropriateness of the intervention components and material; and (3) obtain qualitative process evaluations from observers, CHWs and adolescent females. This study will include the recruitment and training of CHW Teams, and enrol 100 random households with adolescent females from three Agincourt Demographic Surveillance Site villages. These villages will not be included in the planned village cluster randomized trial. Based on the results of the feasibility study, the intervention will be further refined so that it can be evaluated in a cluster randomised controlled trial.

## Conclusions

The paper has described the application of the IM approach to the development of the Ntshembo intervention, and has given an outline of this intervention, as well as described stakeholder consultation in the development process. The IM approach has proved to be useful for ensuring that evidence (both local and international) and theory inform the development of the Ntshembo intervention, and this approach has highlighted the importance of considering personal and environmental determinants of behaviour. It is clear from the evidence presented that it is vital to intervene to address NCD risk and optimise health in SA adolescent girls, particularly pre-pregnancy, in order to reduce the intergenerational transfer of metabolic disease risk. The Ntshembo intervention is novel, both in SA and globally, and could prove to be both scalable and sustainable due to its alignment with health service priorities in SA.

## Competing interests

The authors have no competing interests to declare.

## Authors' contributions

SAN, DD, JMP, KK and SMT conceptualised the project. CED, LKM and SAN completed the IM process and developed the original intervention plan. JMP, KK, SMT and DD provided input on the results of the IM process and intervention plan. CED drafted the manuscript, and all authors commented on and approved the final manuscript.

## Declarations

This work has been funded by the MRC/DfID African Research Leader Scheme.

This article has been published as part of *BMC Public Health* Volume 14 Supplement 2, 2014: Responsive health systems: working with the community on control of non-communicable diseases (NCDs). The full contents of the supplement are available online at http://www.biomedcentral.com/bmcpublichealth/supplements/14/S2.

## References

[B1] Steyn NP Nel JH Parker W Ayah R Mbithe D Urbanisation and the nutrition transition: A comparison of diet and weight status of South African and Kenyan women Scandinavian Journal of Public Health 2012 40 3 229 238 10.1177/1403494812443605 22637361

[B2] Mayosi BM Flisher AJ Lalloo UG Sitas F Tollman SM Bradshaw D The burden of non-communicable diseases in South Africa Lancet 2009 374 9693 934 947 10.1016/S0140-6736(09)61087-4 19709736

[B3] Kimani-Murage EW Kahn K Pettifor JM Tollman SM Dunger DB The prevalence of stunting, overweight and obesity, and metabolic disease risk in rural South African children BMC Public Health 2010 10 158 10.1186/1471-2458-10-158 20338024 PMC2853509

[B4] Abrahams Z Mchiza Z Steyn NP Diet and mortality rates in Sub-Saharan Africa: stages in the nutrition transition BMC Public Health 2011 11 801 10.1186/1471-2458-11-801 21995618 PMC3209469

[B5] Hanson M Gluckman P Developmental origins of noncommunicable disease: population and public health implications The American Journal of Clinical Nutrition 2011 94 6 Suppl 1754S 1758S 21525196 10.3945/ajcn.110.001206

[B6] Hanson MA Gluckman PD Ma RCW Matzen P Biesma RG Early life opportunities for prevention of diabetes in low and middle income countries BMC Public Health 2012 12 1025 10.1186/1471-2458-12-1025 23176627 PMC3526388

[B7] Shisana O Labadarios D Rehle T Simbayi L Zuma K Dhansay A Reddy P Parker W Hoosain E Naidoo P South African National Health and Nutrition Examination Survey (SANHANES-1) 2013 Cape Town: HSRC Press

[B8] Battista M-C Hivert M-F Duval K Baillargeon J-P Intergenerational Cycle of Obesity and Diabetes: How Can We Reduce the Burdens of These Conditions on the Health of Future Generations? Experimental Diabetes Research 2011 2011 10.1155/2011/596060 PMC3205776 22110473

[B9] Vellinga A Zawiejska A Harreiter J Buckley B D Cianni G Lapolla A Corcoy R Simmons D Adelantado JM Damm P Associations of body mass index (maternal BMI) and gestational diabetes mellitus with neonatal and maternal pregnancy outcomes in a multicentre European database (diabetes and pregnancy vitamin D and lifestyle intervention for gestational diabetes mellitus prevention) ISRN Obesity 2012 2012 10.5402/2012/424010 PMC3914269 24527262

[B10] Lindsay RS Gestational diabetes: causes and consequences British Journal of Diabetes and Vascular Disease 2009 9 1 27 31 10.1177/1474651408101644

[B11] Wahabi HA Alzeidan RA Esmaeil SA Pre-pregnancy care for women with pre-gestational diabetes mellitus: a systematic review and meta-analysis BMC Public Health 2012 12 792 10.1186/1471-2458-12-792 22978747 PMC3575330

[B12] Dean S Rudan I Althabe F Webb Girard A Howson C Langer A Lawn J Reeve ME Teela KC Toledano M Setting research priorities for preconception care in low- and middle-income countries: aiming to reduce maternal and child mortality and morbidity PLOS Medicine 2013 10 9 e1001508 10.1371/journal.pmed.1001508 24019762 PMC3760783

[B13] Crozier SR Robinson SM Borland SE Godfrey KM Cooper C Inskip HM SWS Study Group Do women change their health behaviours in pregnancy? Findings from the Southampton Women's Survey Paediatric and Perinatal Epidemiology 2009 23 5 446 453 10.1111/j.1365-3016.2009.01036.x 19689495 PMC3091015

[B14] Kahn K Collinson MA Gomez-Olive FX Mokoena O Twine R Mee P Afolabi SA Clark BD Kabudula CW Khosa A Profile: Agincourt Health and Socio-demographic Surveillance System International Journal of Epidemiology 2012 41 4 988 1001 10.1093/ije/dys115 22933647 PMC3429877

[B15] Bhutta ZA Das JK Rizvi A Gaffey MF Walker N Horton S Webb P Lartey A Black RE The Lancet Nutrition Interventions Review Group Evidence-based interventions for improvement of maternal and child nutrition: what can be done and at what cost? Lancet 2013 382 9890 452 477 10.1016/S0140-6736(13)60996-4 23746776

[B16] Gore FM Bloem PJN Patton GC Ferguson J Joseph V Coffey C Sawyer SM Mathers CD Global burden of disease in young people aged 10-24 years: a systematic analysis Lancet 2011 377 9783 2093 2102 10.1016/S0140-6736(11)60512-6 21652063

[B17] Patton GC Coffey C Cappa C Currie D Riley L Gore F Degenhardt L Richardson D Astone N Sangowawa AO Health of the world's adolescence: a synthesis of internationally comparable data Lancet 2012 379 9826 1665 1675 10.1016/S0140-6736(12)60203-7 22538181

[B18] Reddy SP Resnicow K James S Funani IN Kambaran NS Omardien RG Masuka P Sewpaul R Vaughan RD Mbewu A Rapid increases in overweight and obesity among South African adolescents: comparison of data from the South African National Youth Risk Behaviour Survey in 2002 and 2008 American Journal of Public Health 2012 102 2 262 268 10.2105/AJPH.2011.300222 21940919 PMC3483977

[B19] Jewkes R Vundule C Maforah F Jordaan E Relationship dynamics and teenage pregnancy in South Africa Social Science and Medicine 2001 52 5 733 744 10.1016/S0277-9536(00)00177-5 11218177

[B20] Sawyer SM Afifi RA Bearinger LH Blakemore S-J Dick B Ezeh AC Patton GC Adolescence: a foundation for future health Lancet 2012 379 9826 1630 1640 10.1016/S0140-6736(12)60072-5 22538178

[B21] Viner RM Ozer EM Denny S Marmot M Resnick M Fatusi A Currie C Adolescence and the social determinants of health Lancet 2012 379 9826 1641 1652 10.1016/S0140-6736(12)60149-4 22538179

[B22] Collinson MA Tollman SM Kahn K Clark SJ Garenne M Tienda M, Findley SE, Tollman SM, Preston-Whyte E Highly prevalent circular migration: households, mobility and economic status in rural South Africa Africa on the move: African migration and urbanisation in comparative perspective 2006 Johannesburg: Wits University Press

[B23] Gomez-Olive FX Angotti N Houle B Klipstein-Grobusch K Kabudula C Menken J Williams J Tollman S Clark SJ Prevalence of HIV among those 15 and older in rural South Africa AIDS Care 2013 10.1080/09540121.2012.750710 PMC3778517 23311396

[B24] Bartholomew LK Parcel GS Kok G Gottlieb NH Planning health promotion programs: an Intervention Mapping approach 2006 2 San Fransisco: Jossey-Bass

[B25] Craig P Dieppe P Macintyre S Mitchie S Nazareth I Petticrew M Developing and evaluating complex interventions: the new Medical Research Council Guidance British Medical Journal 2008 337 a1655 10.1136/bmj.a1655 18824488 PMC2769032

[B26] Decat P Nelson E De Meyer S Jaruseviciene L Orozco M Segura Z Gorter A Vega B Cordova K Maes L Community embedded reproductive health interventions for adolescents in Latin America: development and evaluation of a complex multi-centre intervention BMC Public Health 2013 13 1 31 10.1186/1471-2458-13-31 23311647 PMC3599131

[B27] Krolner R Suldrup Jorgensen T Aarestrup A Hjollund Christiansen A Christensen A Due P The Boost study: design of a school- and community-based randomised trial to promote fruit and vegetable consumption among teenagers BMC Public Health 2012 12 1 191 10.1186/1471-2458-12-191 22413782 PMC3375189

[B28] Lloyd JJ Logan S Greaves CJ Wyatt KM Evidence, theory and context - using intervention mapping to develop a school-based intervention to prevent obesity in children International Journal of Behavioral Nutrition and Physical Activity 2011 8 73 10.1186/1479-5868-8-73 21752261 PMC3152876

[B29] Munir F Kalawsky K Wallis D Donaldson-Feilder E Using intervention mapping to develop a work-related guidance tool for those affected by cancer BMC Public Health 2013 13 1 6 10.1186/1471-2458-13-6 23289708 PMC3585779

[B30] Verbestel V De Henauw S Maes L Haerens L Marild S Eiben G Lissner L Moreno LA Lascorz Frauca N Barba G Using the intervention mapping protocol to develop a community-based intervention for the prevention of childhood obesity in a multi-centre European project: the IDEFICS intervention International Journal of Behavioral Nutrition and Physical Activity 2011 8 82 10.1186/1479-5868-8-82 21806806 PMC3169445

[B31] Viester L Verhagen E Proper K van Dongen J Bongers P van der Beek A VIP in construction: systematic development and evaluation of a multifaceted health programme aiming to improve physical activity levels and dietary patterns among construction workers BMC Public Health 2012 12 1 89 10.1186/1471-2458-12-89 22289212 PMC3280176

[B32] Richter LM Norris S Pettifor J Yach D Cameron N Cohort Profile: Mandela's children: the 1990 Birth to Twenty study in South Africa International Journal of Epidemiology 2007 36 3 504 511 10.1093/ije/dym016 17355979 PMC2702039

[B33] Willan S A review of teenage pregnancy in South Africa: experiences of school, and knowledge and access to sexual and repreoductive health services Partners in Sexual Health 2013

[B34] Jewkes R Morrell R Christofides N Empowering teenagers to prevent pregnancy: lessons from South Africa Culture, Health and Sexuality: An International Journal for Research, Intervention and Care 2009 11 7 675 688 10.1080/13691050902846452 19459086

[B35] Reddy SP James S Sewpaul R Koopman F Funani NI Sifunda S Josie J Masuka P Kambaran NS Omardien RG Umthente Uhlaba Usamila – The South African Youth Risk Behaviour Survey 2008 2010 Cape Town

[B36] Edin K 'Ntshembo': Cultural beliefs and practices regarding adolescent lifestyle, pregnancy, delivery and infant care. A qualitative study in rural Mpumalanga Province 2012

[B37] Peltzer K Parker W Mabaso M Makonko E Zuma K Ramlagan S Impact of National HIV and AIDS communication campaigns in South Africa to reduce HIV risk behaviour Scientific World Journal 2012 2012 10.1100/2012/384608 PMC3504395 23213285

[B38] http://www.who.int/mediacentre/factsheets/fs355/en/

[B39] Feeley A Pettifor J Norris S Fast-food consumption among 17-year-olds in the Birth to Twenty cohort South African Journal of Clinical Nutrition 2009 22 3 118 123

[B40] Kimani-Murage EW Kahn K Pettifor JM Tollman SM Klipstein-Grobusch K Norris SA Predictors of adolescent weight status and central obesity in rural South Africa Public Health Nutrition 2011 14 6 1114 1122 10.1017/S1368980011000139 21356151 PMC3370923

[B41] Peer N Bradshaw D Laubscher R Steyn N Steyn K Urban-rural and gender differences in tobacco and alcohol use, diet and physical activity among young black South Africans between 1998 and 2003 Global Health Action 2013 6 19216 23364100 10.3402/gha.v6i0.19216 PMC3559753

[B42] Micklesfield LK Lambert EV Hume DJ Chantler S Pienaar PR Dickie K Puoane T Goedecke JH Socio-cultural, environmental and behavioral determinants of obesity in black South African women Cardiovascular Journal of Africa 2013 24 online publication 10.5830/CVJA-2013-069 PMC3896104 24051701

[B43] Caradas AA Lambert EV Charlton KE An ethnic comparison of eating attitudes and associated body image concerns in adolescent South African schoolgirls Journal of Human Nutrition and Dietetics 2001 14 2 111 120 10.1046/j.1365-277X.2001.00280.x 11330260

[B44] Gitau TM Micklesfield LK Pettifor JM Norris SA Ethnic differences in eating attitudes, body image and self-esteem among adolescent females living in urban South Africa African Journal of Psychiatry in press

[B45] Szabo CP Allwood CW Body figure preference in South African adolescent females: a cross cultural study African Health Sciences 2006 6 4 201 206 17604508 10.5555/afhs.2006.6.4.201 PMC1832064

[B46] Mchiza ZJ Goedecke JH Lambert EV Intra-familial and ethnic effects on attitudinal and perceptual body image: a cohort of South African mother-daughter dyads BMC Public Health 2011 11 433 10.1186/1471-2458-11-433 21645339 PMC3138457

[B47] Gerards SMPL Sleddens EFC Dagnelie PC De Vries NK Kremers SPJ Interventions addressing general parenting to prevent or treat childhood obesity International Journal of Pediatric Obesity 2011 6 2-2 e28 e45 10.3109/17477166.2011.575147 21657977

[B48] Venter I Winterbach A Dietary fat knowledge and intake of mid-adolescents attending public schools in the Bellville / Durbanville area of the city of Cape Town South African Journal of Clinical Nutrition 2010 23 2 75 83

[B49] Pedro TM MacKeown JM Norris SA Variety and total number of food items recorded by a true longitudinal group of urban black South African children at five interceptions between 1995 and 2003 Public Health Nutrition 2008 11 6 616 623 17894914 10.1017/S1368980007000936 PMC2709964

[B50] Sedibe MH Griffiths PL Doak CM Feeley AB Voorend C Norris SA Narratives of urban female adolescents in South Africa: dietary and physical activity practices in an obesogenic environment South African Journal of Clinical Nutrition in press

[B51] Temple NJ Steyn NP Myburgh NG Nel JH Food items consumed by students attending schools in socioeconomic areas in Cape Town, South Africa Nutrition 2006 22 3 252 258 10.1016/j.nut.2005.07.013 16500552

[B52] Sedibe MH Kahn K Edin K Muthoni T Ivarsson A Norris SA Narratives of facilitiators and barriers to healthy eating practices and physical activity among adolescent girls in rural South Africa 2013 10.1186/1471-2431-14-211 PMC4150418 25164604

[B53] van Zyl MK Steyn NP Marais ML Characteristics and factors influencing fast food intake of young adult consumers in Johannesburg, South Africa South African Journal of Clinical Nutrition 2010 23 3 124 130

[B54] Cislak A Safron M Pratt M Gaspar T Luszczynska A Family-related predictors of body weight and weight-related behaviours among children and adolescents: a systematic review Child: Care, Health and Development 2012 38 3 321 331 10.1111/j.1365-2214.2011.01285.x 21752064

[B55] Neumark-Sztainer D Preventing the broad spectrum of weight-related problems: working with parents to help teens achieve a healthy weight and a positive body image Journal of Nutrition Education and Behavior 2005 37 Suppl 2 S133 S139 16246282 10.1016/s1499-4046(06)60214-5

[B56] Pearson N Biddle SJH Gorely T Family correlates of fruit and vegetable consumption in children and adolescents: a systematic review Public Health Nutrition 2009 12 2 267 283 10.1017/S1368980008002589 18559129

[B57] Wang Y Beydoun M Li J Liu Y Moreno LA Do children and their parents eat a similar diet? Resemblance in child and parent dietary intake: a systematic review and meta-analysis Journal of Epidemiology and Community Health 2011 65 2 177 189 10.1136/jech.2009.095901 21051779 PMC3010265

[B58] Savage JS Fisher JO Birch LL Parenting influence on eating behavior: conception to adolescence Journal of Law, Medicine and Ethics 2007 35 1 22 34 10.1111/j.1748-720X.2007.00111.x PMC2531152 17341215

[B59] Sleddens EFC Gerards SMPL Thijs C De Vries NK Kremers SPJ General parenting, childhood overweight and obesity-inducing behaviors: a review International Journal of Childhood Obesity 2011 6 2-2 e12 e27 10.3109/17477166.2011.566339 21657834

[B60] Neumark-Sztainer D Eating among teens: do family mealtimes make a difference for adolescents' nutrition? New Directions for Child and Adolescent Development 2006 111 91 105 16646501 10.1002/cd.157

[B61] Stockmyer C Remember when mom wanted you home for dinner? Nutrition Reviews 2001 59 2 57 60 11310778 10.1111/j.1753-4887.2001.tb06978.x

[B62] Krolner R Rasmussen M Brug J Klepp KI Wind M Due P Determinants of fruit and vegetable consumption among children and adolescents: a review of the literature. Part II: qualitative studies International Journal of Behavioral Nutrition and Physical Activity 2011 8 112 10.1186/1479-5868-8-112 21999291 PMC3260149

[B63] Dumith SC Gigante DP Domingues MR Hallal PC Menezes AMB A longitudional evaluation of physical activity in Brazilian adolescents: tracking, change and predictors Pediatric Exercise Science 2012 24 1 58 71 22433265 10.1123/pes.24.1.58 PMC3650301

[B64] Nader PR Bradley RH Houts RM McRitchie SL O’Brien M Moderate-to-vigorous physical activity from ages 9 to 15 years Journal of the American Medical Association 2009 300 3 295 305 18632544 10.1001/jama.300.3.295

[B65] Olds T Wake M Patton G Ridley K Waters E Williams J Hesketh K How do school-day activity patterns differ with age and gender across adolescence? Journal of Adolescent Health 2009 44 1 64 72 10.1016/j.jadohealth.2008.05.003 19101460

[B66] Dollman J Maher C Olds TS Ridley K Physical activity and screen time behaviour in metropolitan, regional and rural adolescents: a cross-sectional study of Australians aged 9-16 years Journal of Science and Medicine in Sport 2012 15 1 32 37 10.1016/j.jsams.2011.05.011 21742553

[B67] Carver A Timperio AF Hesketh KD Ridgers ND Salmon JL Crawford DA How is active transport associated with children’s and adolescents’ physical activity over time? International Journal of Behavioral Nutrition and Physical Activity 2011 8 126 10.1186/1479-5868-8-126 22081977 PMC3226569

[B68] Dumith SC Domingues MR Gigante DP Hallal PC Menezes AMB Kohl HW Prevalence and correlates of physical activity among adolescents from Southern Brazil Revista de Saude Publica 2010 44 3 457 467 10.1590/S0034-89102010000300009 20549018

[B69] Mota J Gomes H Almeida M Ribeiro JC Carvalho J Santos MP Active versus passive transportation to school – differences in screen time, socio-economic position and perceived environmental characteristics in adolescent girls Annals of Human Biology 2007 34 3 273 282 10.1080/03014460701308615 17612859

[B70] Micklesfield LK Pedro TM Kahn K Kinsman J Pettifor JM Tollman SM Norris SA Physical activity and sedentary behavior among adolescents in rural South Africa: levels, patterns and determinants In review 10.1186/1471-2458-14-40 PMC3897951 24433276

[B71] Uijtdewilligen L Nauta J Singh AS van Mechelen W Twisk JWR van der Horst K Chinapaw MJM Determinants of physical activity and sedentary behaviour in young people: a review and quality synthesis of prospective studies British Journal of Sports Medicine 2011 45 11 898 905 10.1136/bjsports-2011-090197 21836173

[B72] Dumith SC Gigante DP Domingues MR Hallal PC Menezes AMB Kohl HW Predictors of physical activity change during adolescence: a 3.5 years follow-up Public Health Nutrition 2012 15 12 2237 2245 10.1017/S1368980012000948 22464063 PMC3501818

[B73] Gordon-Larsen P McMurray RG Popkin BM Determinants of adolescent physical activity and inactivity patterns Pediatrics 2000 105 4 e83 10835096 10.1542/peds.105.6.e83

[B74] van der Horst K Chinapaw MJ Twisk JWR van Mechelen W A brief review on correlates on physical activity and sedentariness in youth Medicine and Science in Sports and Exercise 2007 39 8 1241 1250 10.1249/mss.0b013e318059bf35 17762356

[B75] Kinsman J Norris SA Kahn K Twine R Riggle K Mathebula J Ngobeni S Monareng N Edin K Micklesfield LK A model for promoting physical activity among South African adolescent girls In review 10.3402/gha.v8.28790 PMC4684577 26685095

[B76] Hardy LL Bass SL Booth ML Changes in sedentary behaviour among adolescent girls: a 2.5 years prospective cohort study Journal of Adolescent Health 2007 40 2 158 165 10.1016/j.jadohealth.2006.09.009 17259056

[B77] Tremblay MS LeBlanc AG Kho ME Saunders TJ Larouche R Colley RC Goldfield G Gorber SC Systematic review of sedentary behaviour and health indicators in school-aged children and youth International Journal of Behavioral Nutrition and Physical Activity 2011 8 98 10.1186/1479-5868-8-98 21936895 PMC3186735

[B78] Abraham C Michie S A taxonomy of behavior change techniques used in interventions Health Psychology 2008 27 3 379 387 18624603 10.1037/0278-6133.27.3.379

[B79] Bandura A Social foundations of thought and action: a social cognitive theory 1986 Englewood Cliffs, NJ: Prentice-Hall

[B80] Ajzen I The theory of planned behavior Organizational Behavior and Human Decision Processes 1991 50 2 179 211 10.1016/0749-5978(91)90020-T

[B81] Carver CS Scheier MF Control theory: a useful conceptual framework for personality-social, clinical, and health psychology Psychological Bulletin 1982 92 1 111 135 7134324

[B82] Fisher JD Fisher WA Changing AIDS-risk behavior Psychological Bulletin 1992 111 3 455 474 1594721 10.1037/0033-2909.111.3.455

[B83] Hawkes SJ Gomez GB Broutet N Early antenatal care: does it make a difference to outcomes of pregnancy associated with syphilis? A systematic review and meta-analysis PLoS One 2013 8 2 e56713 10.1371/journal.pone.0056713 23468875 PMC3585307

[B84] Oakley L Gray R Kurinczuk JJ Brocklehurst P Hollowell J A systematic review of the effectiveness of interventions to increase the early initiation of antenatal care in socially disadvantaged and vulnerable women 2009 10.1186/1471-2393-11-13 PMC3050773 21314944

[B85] Hollowell J Oakley L Vigurs C Barnett-Page E Kavanagh J Oliver S Increasing the early initiation of antenatal care by Black and Minority Ethnic women in the United Kingdom: a systematic review and mixed methods synthesis of women’s views and the literature on intervention effectiveness 2012

[B86] Grydeland M Bergh IH Bjelland M Lien N Andersen LF Ommundsen Y Klepp KI Anderssen SA Intervention effects on physical activity: the HEIA study – a cluster randomized controlled trial International Journal of Behavioral Nutrition and Physical Activity 2013 10 17 10.1186/1479-5868-10-17 23379535 PMC3598379

[B87] Lubans DR Morgan PJ Okely AD Dewar D Collins CE Batterham M Callister R Plotnikoff RC Preventing obesity among adolescent girls: one-year outcomes of the Nutrition and Enjoyable Activity for Teen Girls (NEAT Girls) cluster randomized controlled trial Archives of Pediatrics and Adolescent Medicine 2012 166 9 821 827 10.1001/archpediatrics.2012.41 22566517

[B88] Singh AS Chinapaw MJM Brug J van Mechelen W Dutch obesity intervention in teenagers: effectiveness of a school-based program on body composition and behavior Archives of Pediatrics and Adolescent Medicine 2009 163 4 309 317 10.1001/archpediatrics.2009.2 19349559

[B89] Flodmark CE Marcus C Britton M Interventions to prevent obesity in children and adolescents: a systematic review International Journal of Obesity 2006 30 4 579 589 10.1038/sj.ijo.0803290 16570086

[B90] Cliff DP Okely AD Morgan PJ Jones RA Steele JR The impact of child and adolescent obesity treatment interventions on physical activity: a systematic review Obesity Reviews 2010 11 7 516 530 19656311 10.1111/j.1467-789X.2009.00625.x

[B91] Millar L Kremer P de Silva-Sanigorski A McCabe MP Mavoa H Moodie M Utter J Bell C Malakellis M Mathews L Reduction in overweight and obesity from a 3-year community-based intervention in Australia: the ‘It’s Your Move!’ project Obesity Reviews 2011 12 Supplement 2 20 28 22008556 10.1111/j.1467-789X.2011.00904.x

[B92] Nguyen B Shrewsbury V O’Connor J Steinbeck KS Lee A Hill AJ Shah S Kohn MR Torvaldsen S Baur LA Twelve-month outcomes of the Loozit randomized controlled trial Archives of Pediatrics and Adolescent Medicine 2012 166 2 170 177 10.1001/archpediatrics.2011.841 22312175

[B93] Fotu KF Moodie MM Mavoa HM Pomana S Schultz JT Swinburn BA Process evaluation of a community-based adolescent obesity prevention project in Tonga BMC Public Health 2011 11 284 10.1186/1471-2458-11-284 21549018 PMC3098171

[B94] Khambalia AZ Dickinson S Hardy LL Baur LA A synthesis of existing systematic reviews and meta-analyses of school-based behavioural interventions for controlling and preventing obesity Obesity Reviews 2012 13 3 214 233 10.1111/j.1467-789X.2011.00947.x 22070186

[B95] Niemeier BS Hektner JM Enger KB Parent participation in weight-related health interventions for children and adolescents: a systematic review and meta-analysis Preventive Medicine 2012 55 1 3 13 10.1016/j.ypmed.2012.04.021 22575353

[B96] de Mattia L Lemont L Meurer L Do interventions to limit sedentary behaviours change behaviour and reduce childhood obesity? A critical review of the literature Obesity Reviews 2007 8 1 69 81 10.1111/j.1467-789X.2006.00259.x 17212797

[B97] Jewkes R Abrahams N Mvo Z Why do nurses abuse patients? Reflections from South Africa obstetric services Social Science and Medicine 1998 47 11 1781 1795 10.1016/S0277-9536(98)00240-8 9877348

[B98] Wood K Jewkes R Blood blockages and scolding nurses: barriers to adolescent contraceptive use in South Africa Reproductive Health Matters 2006 14 27 109 118 10.1016/S0968-8080(06)27231-8 16713885

[B99] Baloyi GO The evaluation of the National Adolescent-Friendly Clinic Initiative (NAFCI) programme in Greater Tzaneen sub-district, Limpopo Province, South Africa 2006 University of South Africa

[B100] Dickson KE Ashton J Smith JM Does setting adolescent-friendly standards improve the quality of care in clinics? Evidence from South Africa International Journal for Quality in Health Care 2007 19 2 80 89 10.1093/intqhc/mzl070 17277012

[B101] Geary R Young people's experiences of youth friendly health services Unpublished thesis 2013

[B102] Schriver BL “They’re treating us bad now, never the same.” Young people’s perceptions of health services in Soweto, South Africa: a qualitative investigation Unpublished thesis 2013

[B103] Koon AD Goudge J Norris SA A systematic review of generalist and specialist community health workers for delivering adolescent health services in sub-Saharan Africa Human Resources for Health 2013 11 54 10.1186/1478-4491-11-54 24160988 PMC3874771

[B104] Barker M Baird J Lawrence W Jarman M Black C Barnard K Cradock S Davies J Margetts B Inskip H The Southampton Initiative for Health : a complex intervention to improve the diets and increase the physical activity levels of women from disadvantaged communities Journal of Health Psychology 2011 16 1 178 191 10.1177/1359105310371397 20709878 PMC3685267

[B105] Tinati T Lawrence W Ntani G Black C Cradock S Jarman M Pease A Begum R Inskip H Cooper C Implementation of new Healthy Conversation Skills to support lifestyle changes – what helps and what hinders? Experiences of Sure Start Children’s Centre staff Health and Social Care in the Community 2012 20 4 430 437 10.1111/j.1365-2524.2012.01063.x 22452549 PMC3679516

